# Learning motivations and effort beliefs in Confucian cultural context: A dual-mode theoretical framework of achievement goal

**DOI:** 10.3389/fpsyg.2023.1058456

**Published:** 2023-02-02

**Authors:** Shun-Wen Chen

**Affiliations:** Department of Educational Psychology and Counseling, National Tsing Hua University, Hsinchu, Taiwan

**Keywords:** academic achievements, beliefs about effort, learning virtues, personal goals, role obligation, vertical goals

## Abstract

For decades, results of international academic assessments have shown that students in the Confucian cultural circle performed outstandingly well. However, many studies also showed that East Asian students often experienced high pressure and had low interest in academic learning. The “high achievement but low interest” phenomenon has aroused great interest in psychologists and educators. From the emic perspective of cultural psychology, this theoretical article aims to propose (1) a dual-mode framework of achievement goals to conceptualize the motivation for academic learning and (2) two kinds of effort beliefs (obligation-oriented and improvement-oriented belief about effort) students may develop when pursuing academic achievement in societies influenced by Confucian heritage culture. Moreover, a series of empirical studies based on the framework are presented in this article to show that (1) Chinese students’ academic striving is motivated not only by their interest but also by role obligation or virtue of effort, (2) students’ effort beliefs could predict their learning emotion and behavioral tendency, and (3) students’ effort beliefs could be influenced by their parents’ and teachers’ effort beliefs. The theoretical and practical implications of the framework are discussed.

## 1. Introduction

Students in societies influenced by Confucian heritage culture (including Taiwan, Japan, South Korea, Hong Kong, and Singapore) have frequently demonstrated academic achievements that outshined their Western counterparts in international assessments such as the Trends in International Mathematics and Science Study (TIMSS) and the Program of International Student Assessment (PISA). However, the results of those assessments also showed that East Asian students tended to have low interest in their academic learning (OECD, 2016, 2019; Mullis et al., 2016a,b, 2020). In a similar vein, previous studies have shown that East Asian students often experienced high pressure from social expectation and had low interest in academic learning (Ang and Huan, 2006; Lin and Huang, 2014; Fwu et al., 2018; Ma et al., 2018; Lee et al., 2020). Paradoxically, some experimental and questionnaire researches found that parents’ expectation had positive impact on East Asian learners’ academic motivation, involvement and achievement (Iyengar and Lepper, 1999; D’Ailly, 2003; Sheldon et al., 2004; Li, 2012). Consequently, the “high achievement but low interest” phenomenon and learning motivations of East Asian students arouse great interest in cultural and educational psychologists.

Researchers have indicated that mainstream Western motivation theories could not comprehensively explain East Asian people’s learning motivation because those theories usually emphasized the intrinsic motivation, personal autonomy or beliefs about ability (Iyengar and Lepper, 1999; Grant and Dweck, 2001; Elliott and Bempechat, 2002; Ho and Hau, 2008; King and McInerney, 2016). From the emic perspective of cultural psychology, it is crucial to construct culture-inclusive theories to understand non-Western people’s learning psychology and behavior *via* cultural meaning systems (Shweder, 2000; Hwang, 2019).

Many East Asian societies are culturally influenced by the Confucian tradition, which has a comprehensive value system emphasizing social roles, effort-making, self-cultivation and academic achievements (Ames, 2011; Hwang, 2012; Li, 2012). In Confucian doctrines, a virtuous person should improve oneself and be in accordance with his/her social roles. If one improves or cultivates one’s own self through continuously learning and daily practices, s/he will be regarded as a virtuous person. These cultural values could influence East Asian people’s attitudes and behaviors associated with learning (Chen et al., 2009, 2019; Li, 2012; Peterson et al., 2013; Fwu et al., 2017, 2018). Li (2012) indicated that the conception of “learning” (*xué-xí*) in Chinese is virtue-oriented. A “good” student is one who has the qualities of diligence, earnestness, sincerity, perseverance, steadfastness, and endurance of hardship in learning. These characteristics are all synonymous with “effort” and can be referred to as “learning virtues.” Previous studies have shown that, among students in Hong Kong, Taiwan and Korea, to study hard and to excel in academic performance were considered to be the primary obligations (Hong, 2001; Fwu et al., 2014). Besides, the duty conceptions were strong predictors for Asian students on academic performance (Peterson et al., 2013).

Institutionally, for over 1,000 years (from about 600 CE to 1905 CE), China implemented an “official examination system” (*kē-jǔ*) to select government officials by national exams. This system assessed scholars on their knowledge of Confucian classics and was regarded as a major method of selecting and promoting talented and virtuous persons to be officials. The system in turn instilled values and doctrines of Confucianism into the minds, habits and practices of the general populace for generations. Studying hard to pass these exams and be admitted as officials was an effective way for commoners to raise their social class. Nowadays, to earn a high level of academic degree is still regarded as a matter of great honor for one’s family (Hwang, 2012; Li, 2012).

## 2. Dual-mode framework of achievement goal

In order to conceptualize the motivations for academic pursuit in societies influenced by Confucian traditions, the author proposed a dual-mode framework of achievement goal (see Table 1), revised from the work of Chen et al. (2009). According to the framework, two modes of achievement goal can be differentiated in Confucian cultural context. The *vertical goals* are socially constructed and characterized by high expectations from significant others, such as parents or teachers. Many of these goals are derived by the general public from a set of highly recognized common values; hence, individuals’ performances in the pursuit of these goals will usually be ranked on a vertical ladder of achievements. In order to climb up the “achievement pyramid,” one needs to overcome the difficulties and failures of such goals. Those who work hard on vertical goals tend to be motivated by dutifulness or identity of role obligation and be viewed as virtuous persons, while those who do not may be seen as flawed in character. Typically, academic achievements are usually regarded as vertical goals in societies influenced by Confucian heritage culture. Fwu et al. (2014, 2016, 2017) conducted scenario experiments and administered questionnaires to junior high school students as well as their teachers and parents. The results showed that hard-working students who were successful in pursuit of academic goals tended to win more credit from parents and teachers due to their better moral image.

**Table 1:** The dual-mode framework of achievement goal.

	Personal goals	Vertical goals
Primary source of goal-construction	Autonomous interest	Social expectation
Motivation mode	Intrinsic motivation	Dutifulness
Manifestation of virtues	Insignificant	Significant
Functions of psychosocial adaptation	Maintenance of positive self-regard	Identification of role obligations
Self-attribution pattern	Self-enhancement	Effort model
Implicit beliefs	Ability beliefs: Entity or incremental beliefs of ability	Effort beliefs: Obligation-oriented and improvement-oriented beliefs about effort

On the other hand, the *personal* or *non-vertical goals* are constructed by individuals’ autonomous interests. Individuals may choose and define the content and criteria for their personal goals from a wide variety of domains. Such goals may not have high social value and are not necessarily subject to consistent expectations from significant others. These goals include personal interests or hobbies with intrinsic motivation. Moreover, those who pursue these goals, irrespective of the degree of effort they make, will not be evaluated from the perspective of moral virtues. Previous studies showed that achievements in the arts or sports, for instance, were typical examples of personal goals in Taiwan (Chen et al., 2009; Fwu et al., 2016).

A series of empirical studies based on the dual-mode framework of achievement goal have been conducted. Chen et al. (2009) adopted the scenario stimulation method in two experimental studies to investigate the attributional patterns of different goals. They argued that motivation theories developed in individualistic cultures often emphasize the positive impact of autonomous interest on well-being in the process of pursuing achievements (see Deci and Ryan, 1985, 2000; Ryan and Deci, 2018). This may apply to the case of achieving personal goals; namely, when people pursuing personal goals in daily life, the main psychosocial function involved is to maintain positive self-regard. Therefore, Chen et al. (2009) hypothesized that, when attributing their success or failure in personal goals, individuals tend to exhibit the self-enhancement pattern, that is, to attribute failure to external factors and success to internal factors.

For pursuing vertical goals, however, the main psychosocial function is to form social identities by fulfilling one’s role obligations with highly social expectations. Because it is so important to achieve vertical goals, one cannot afford to allow uncontrollable factors—such as innate ability, luck, or task difficulty—to be the determining ones. People are likely to emphasize effort as a way to prompt oneself and to make more effort for future success, especially in the case of failure. Thus, Chen et al. (2009) hypothesized that individuals tend to adopt the self-enhancement pattern or “effort model,” that is, to attribute their failure to lack of effort. The scenario stimulation method was adopted in two experimental studies. The results showed that, in pursuit of vertical goals such as academic achievements, Taiwanese undergraduates indeed tended to attribute their failure to lack of effort. Nonetheless, in pursuit of personal goals (e.g., participating singing competitions), they tended to attribute success to ability and failure to luck or task difficulty.

Furthermore, according to the dual-mode framework, students’ academic striving is motivated not only by their academic interest but also by identity of role obligation. In order to investigate the hypothesis, Chen and Wei (2013) developed scales to measure 176 collage students’ academic interest, role identity, learning satisfaction and academic engagement. The results showed that students’ role identity could directly predict their academic engagement. However, the effect of their academic interest on academic engagement was indirect and mediated by learning satisfaction. Huang et al. (2015) adopted the same scales to measure 1,132 undergraduates’ role identity, intrinsic motivation and learning engagements in learning foreign languages. The results showed that students’ role identity was positively correlated with their learning engagements in the classroom, whereas intrinsic motivation was positively correlated with learning engagements outside the classroom.

## 3. Effort beliefs towards academic learning in Confucian culture

According to the dual-mode framework of achievement goal, when pursuing vertical goals, people tend to believe that it is one’s duty to exert oneself and that effort-making is the crucial way to improve one’s performance. In other words, pursuing academic achievements is often regarded as student’s role obligation. Thus, when pursuing academic achievements, students may develop two kinds of beliefs about effort (Chen et al., 2016, 2019; Wang and Chen, 2020). One is the obligation-oriented belief about effort (OBE) which is to believe that making effort in learning is a student’s role obligation. The other is the improvement-oriented belief about effort (IBE) which is to believe that effort can conquer one’s limitations and improve one’s academic performance. To put it differently, if one believes that it’s a duty to exert her-or himself (OBE), effort-making is regarded as a moral virtue or purpose. On the other hand, if one believes that making effort is the crucial way to improve performance (IBE), effort-making is regarded as a method or means of achieving goals.

Since it’s important to hold OBE and IBE for achieving academic goals, it’s possible that these effort beliefs may predict students’ learning emotion and striving behaviors. In order to investigate the predictive effects of effort beliefs on academic learning, Chen et al. (2016, 2019) developed an Effort Beliefs Scale to measure people’s OBE and IBE. In one study, 475 undergraduates were asked to report their feelings and behaviors after a real-life event of academic failure (Chen et al., 2019). The results of structural equation models showed that both OBE and IBE could predict the “feeling of indebtedness” after academic failure. The feeling or sense of indebtedness (*kuì jiù*) is a feeling of failure with respect to one’s responsibilities or obligations. If one fails to uphold one’s role obligations, s/he may experience a feeling of indebtedness to others. Feeling of indebtedness is a common emotion with psychosocial function for East Asians (Markus and Kitayama, 1991; Bedford and Hwang, 2003; Chiu, 2011; Kang and Larson, 2014). In the same vein, Fwu et al. (2018) found that students who regarded the pursuit of vertical goals as fulfilling role obligations felt indebted toward their parents and themselves after reflecting upon their academic failure. To get rid of feelings of indebtedness, students tended to motivate themselves to work harder to achieve academic success.

Furthermore, the results of Chen et al. (2019) showed that only OBE could predict students’ striving behaviors after academic failure. It’s worth to note that, in that study, the researchers also measured participants’ implicit belief of ability, a construal purposed by Dweck (2006) and Dweck and Leggett (1988). According to Dweck’s model, when students experience failure, those who hold the entity belief of intelligence or ability may feel helplessness and avoid studying harder. In contrast, those who hold the incremental belief of ability may work hard to learn and face the challenge of failure. However, the results of Chen et al. (2019) showed that the implicit belief of ability was correlated with neither participants’ striving behaviors nor their feelings of indebtedness after their poor learning. Since most studies of Dweck’s model were conducted in Western cultures, whether the model could be generalized to East Asian societies is an issue (Grant and Dweck, 2001). Besides, though Dweck’s dichotomous model takes account of one’s incremental belief about ability, it does not take cognizance of the cultural values among East Asian societies, nor does it identify the psychological construal of obligation-oriented belief about effort.

In addition, it’s possible that individuals develop their effort beliefs through socialization process of family or school. Li (2012) analyzed over 200 recordings of Taiwanese and American mothers talking with their children about learning. The results of content analysis showed that Taiwanese mothers talked most about effort, diligence, and concentration, especially when the topic was the children’s poor learning. However, American mothers and children talked mostly about mental abilities and a positive affect toward learning. Therefore, Wang and Chen (2020) hypothesized that children’s effort beliefs could be influenced by their parents. The Effort Belief Scale was adopted to measure 266 pairs of parents and children in Taiwan. The results of structural equation models and mediating analyses showed that elementary school children’s effort beliefs could be predicted by their parents’ effort beliefs. Moreover, Wang and Chen found that, similar to the result of Chen et al. (2019), only children’s OBE could predict their learning engagements in school.

In order to investigate the relationships between teachers’ effort beliefs and their teaching attitudes, Chen et al. (2016) measured 151 high school teachers’ effort beliefs and their attitudes toward students. The results showed that teachers’ OBE could positively predict their favoritism, praises, and future expectations of struggling students, but negatively predict their favoritism and praises of smart students. Besides, teachers’ IBE could negatively predict their expectations of smart students and favoritism of struggling students, but positively predict their praises of smart students. In sum, the values and beliefs about effort in pursuing vertical goals such as academic achievements could be transmitted from generation to generation *via* the socialization of family and school.

## 4. Discussion

The main purposes of this article is to propose a dual-mode theoretical framework of achievement goal and construes of effort beliefs to understand students’ learning motivations in Confucian cultural context. There are some theoretical contributions of the framework. Firstly, we argue that Chinese students’ striving for learning is motivated not only by academic interest but also by role obligation or virtue of effort. Secondly, when pursuing vertical goals such as academic achievements, individuals are likely to develop obligation-oriented and improvement-oriented beliefs about effort. These beliefs about effort may predict students’ learning emotions and engagements. Thirdly, these effort beliefs may be transmitted from parents and teachers. Moreover, in this article, a series of empirical studies based on the theoretical framework are presented, and the results of those studies support the framework.

All in all, from the perspective of emic approach of cultural psychology (Shweder, 2000; Hwang, 2019), we argue that the meaning system of Confucian heritage culture is crucial for the understanding of learners’ motivation among East Asian societies. Future studies can further investigate various characteristics of vertical or personal goals. Researchers can also explore the social and cultural antecedents of effort beliefs as well as the cognitive, affective and behavioral consequences of these beliefs on academic pursuits.

It is important to note that, because vertical and personal goals are conceptual types, it is not our intention to categorize all goals in real-life situations into two poles of a dichotomy. Although Confucianism places high emphasis on the obligations inherent in people’s social roles, it also recognizes the importance of personal agency and rational reflection (Ames, 2011; Hwang, 2012; Chan, 2014). In many East Asian societies, maintenance of autonomous self and identification with social roles are often regarded as two sides of one coin in the cultural scripts for an ideal personhood and living a meaningful life (Doi, 1986).

For practical implications, if a student would like to integrate intrinsic and extrinsic motivations in academic learning, s/he can attain the goals set by others and try to develop autonomous interest concurrently, or gradually transform vertical goals into personal ones (Carver and Scheier, 2000). However, though students who are not good at academics and still obligate to work hard may fulfill their role obligations, a continuous struggle without improved outcomes may also cause strong frustrations and negative emotions (Fwu et al., 2017). Thus, it is a critical issue for educators to find a remedy to ease the negative impacts while retaining the positive effects of effort beliefs on learning motivation in societies influenced by Confucian heritage culture.

## Data availability statement

The original contributions presented in the study are included in the article/supplementary material, further inquiries can be directed to the corresponding author.

## Author contributions

The author confirms being the sole contributor of this work and has approved it for publication.

## Conflict of interest

The author declares that the research was conducted in the absence of any commercial or financial relationships that could be construed as a potential conflict of interest.

## Publisher’s note

All claims expressed in this article are solely those of the authors and do not necessarily represent those of their affiliated organizations, or those of the publisher, the editors and the reviewers. Any product that may be evaluated in this article, or claim that may be made by its manufacturer, is not guaranteed or endorsed by the publisher.
